# Systematic Literature Review on Visual Analytics of Predictive Maintenance in the Manufacturing Industry

**DOI:** 10.3390/s22176321

**Published:** 2022-08-23

**Authors:** Xiang Cheng, Jun Kit Chaw, Kam Meng Goh, Tin Tin Ting, Shafrida Sahrani, Mohammad Nazir Ahmad, Rabiah Abdul Kadir, Mei Choo Ang

**Affiliations:** 1 Institute of IR4.0, Universiti Kebangsaan Malaysia (UKM), Bangi 43600, Selangor, Malaysia; 2 Department of Electrical and Electronics Engineering, Faculty of Engineering and Technology, Tunku Abdul Rahman University College, Kampus Utama, Jalan Genting Kelang, Kuala Lumpur 53300, Malaysia; 3 Faculty of Data Science and Information Technology, INTI International University, Nilai 71800, Negeri Sembilan, Malaysia

**Keywords:** predictive maintenance (PdM), visual analytics, industry 4.0, machine learning, deep learning, explainable artificial intelligence

## Abstract

The widespread adoption of cyber-physical systems and other cutting-edge digital technology in manufacturing industry production facilities may motivate stakeholders to embrace the idea of Industry 4.0. Some industrial companies already have different sensors installed on their machines; however, without proper analysis, the data collected is not useful. This systematic review’s main goal is to synthesize the existing evidence on the application of predictive maintenance (PdM) with visual aids and to identify the key knowledge gaps in areas including utilities, power generation, industry, and energy consumption. After a thorough search and evaluation for relevancy, 37 documents were identified. Moreover, we identified the visual analytics of PdM, including anomaly detection, planning/scheduling, exploratory data analysis (EDA), and explainable artificial intelligence (XAI). The findings revealed that anomaly detection was a major domain in PdM-related works. We conclude that most of the literature lacks depth in terms of an overall framework that combines data-driven and knowledge-driven techniques of PdM in the manufacturing industry. Some works that utilized both techniques indicated promising results, but there is insufficient research on involving maintenance personnel’s feedback in the latter stage of PdM architecture. Thus, there are still pertinent issues that need to be investigated, and limitations that need to be overcome before PdM is deployed with minimal human involvement.

## 1. Introduction

In the past decades, the nine pillars of Industry 4.0 that encompass cyber-physical systems, the Internet of Things, big data, 3D printing, robotics, simulation, augmented reality, cloud computing, and cyber security have enabled machines to solve problems with autonomous and self-regulating systems. By applying suitable artificial intelligence, the industry can reduce factory downtime, optimize production, and achieve better energy management [1].

With the advancement of Industry 4.0, predictive maintenance (PdM) has become prevalent in the manufacturing industry with the availability of different equipment and “smart” (internet-connected) measurement sensors, including temperature measurement sensors or smart power meters, that collect all data directly from the field. Moreover, the growing awareness of sustainability, resource, and energy efficiency has expedited the implementation of PdM in many advanced countries. Nevertheless, the manufacturing industry is still hesitant to modernize its infrastructure and legacy systems. 

The proliferating deployment of cyber-physical systems and other advanced digital technologies in the production facilities of the manufacturing industry could encourage the stakeholders to invest in PdM. These technologies facilitate the collection of large volumes of digital data via vibration sensors, acoustic sensors, temperature sensors, power consumption sensors, and thermal cameras. Thus, the condition of machinery and equipment could be monitored. Any maintenance tasks could be triggered earlier if issues are detected. 

Different approaches for predicting or forecasting energy consumption in the manufacturing industry have been proposed over the past 25 years [2]. Walther et al. classified the approaches into seven categories, system boundary, modeling technique, modeling focus, modeling horizon, modeling perspective, modeling purpose, and model output. The authors pointed out that there is no standardized procedure to compare the results from different modeling techniques. However, it may not be necessary, as different manufacturing industries may have different operating procedures. The model built should also be task-oriented instead of too generalized to other data that are not relevant. 

Many research works have demonstrated promising results in detecting anomalous energy consumption behaviors using artificial intelligence (AI) [3]. For example, various machine learning models were investigated to enhance energy efficiency in common households [4,5]. Most of the efforts were made to detect abnormal power consumption, which was often related to the malfunction of appliances. The consumption of each appliance is different and varies from person to person. More so in the manufacturing industry, where machine failures can cause downtime that affects the overall success of the company [6].

Anomaly detection in either high power consumption or sudden changes in machine temperature allows AI to predict the behavior of individual systems in the production process. In [6,7], the authors showed that anomaly detectors could be installed by using existing electric infrastructures; however, this might involve high implementation costs. Their solutions were built upon machine learning methods. Another common denominator of both studies is that the data are acquired from the production process, so the PdM can be targeted to a specific machine. One popular machine learning method, the neural networks model, was used for anomaly detection in [8,9]. Currently, companies focus on the continuity of production and decreased downtime caused by machine failures. Most of these problems can be solved by embracing the Industry 4.0 concept, which includes PdM [1,10]. 

Although the Industry 4.0 concept is promising, domain experts in the manufacturing industry are typically not familiar with advanced AI tools. Data visualization tools were developed to optimize the personnel’s decision-making process [11,12]. Due to the lack of annotated power consumption datasets for specific machines to train, validate, and test the machine learning model, it is essential to have a subjective and interactive visualization of power consumption patterns to interpret any anomaly better [3]. 

**Figure 1:**
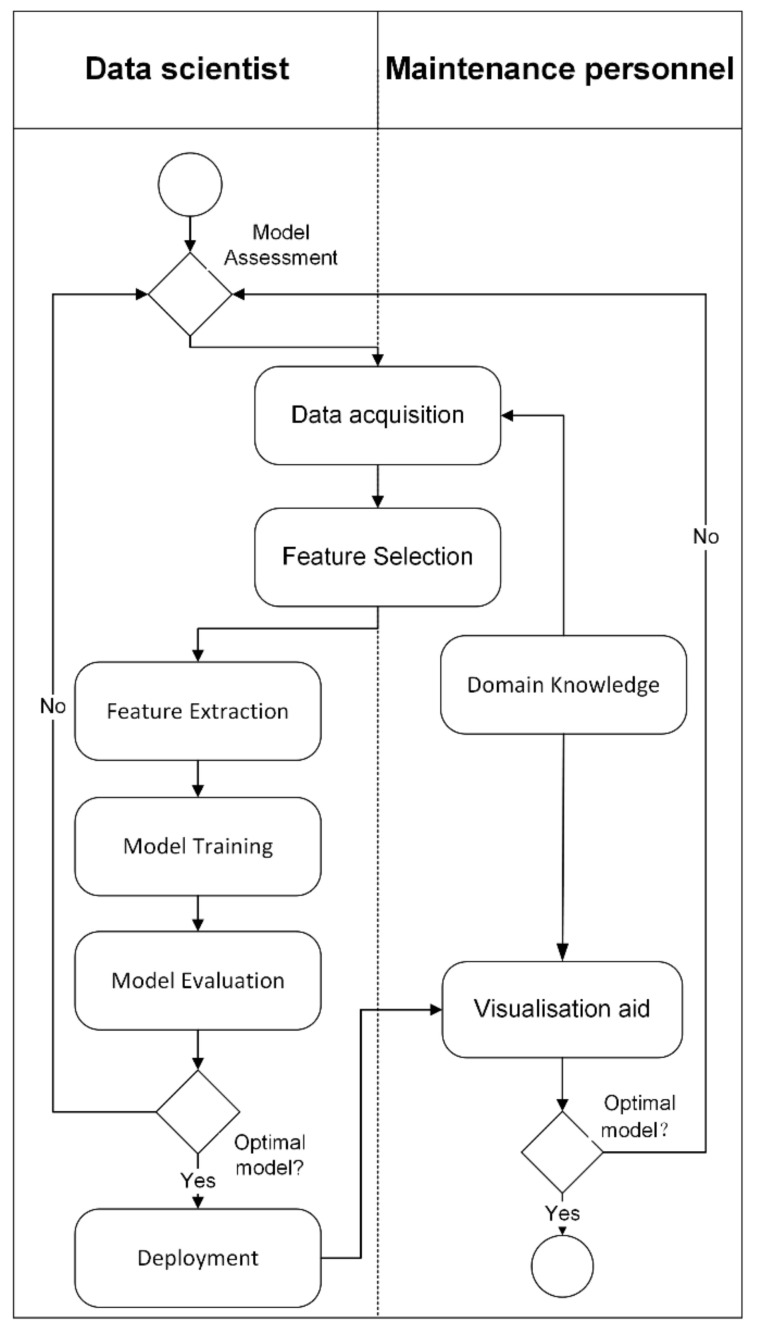
Process flow to set up PdM architecture.

Overall, machine learning shows promising opportunities to effectively resolve anomaly detection and, subsequently, help in PdM. To perform more effective preventive measures, a visualization tool is required for domain experts to better interpret the data patterns. Generally, the process of setting up a PdM architecture should integrate both the domain knowledge of data analysts and maintenance personnel, as illustrated in Figure 1.

For example, Antonio L. Alfeo et al. [13] and Wenjin Yu [14] proposed the PdM architecture comprehensively from data acquisition until deployment with continuous improvement. In terms of data acquisition, the former obtained the temperature and vibration signals in the form of time series data from operation to failure through sensors installed at the experimental platform Pronostia [15], which were publicly accessible; while the latter collected structured and unstructured data from different data sources that were sent to a central cluster in a real-time setting. Both approaches identified machines that required maintenance and determined conditions that could lead to downtime. The approach in [13] found that in a degrading bearing, the acceleration of the vibration signal would be regularly equal to or greater than 1 g. One of the unsupervised anomaly detection methods most frequently used in the manufacturing industry is K-means clustering [14]. The PdM employed aimed to allow the engineer to have enough time to find a solution between the intervals of anomaly detected and the actual incident. The predictive model was expected to give information about the expected downtime or degradation of specific machines. However, the model may detect anomalies in small intervals that could be regarded as noise. Thus, feedback from domain experts is necessary to enhance the model. Instead of dismissing these discovered results as false positives, the domain experts would further examine the data acquired from the machine to determine if there were any irregularities during the time when anomalies were detected. Nevertheless, when domain knowledge is incorporated with PdM, the learned model is more interpretable and reliable with respect to common knowledge. The existing approaches mostly reported that it is challenging to discover representative and contributing features for PdM and primarily depends on the domain experts. Although initially, the training time to adopt PdM is a high management cost, the end results are rewarding, as studies have proven that PdM could prevent more serious events from happening, i.e., unexpected machine downtime which could affect the production yield. In fact, PdM needs to define different maintenance alternatives for different equipment. Hence, the maintenance problem can be identified as a combinatorial optimization problem. This implies the presence of a function with different optimal criteria depending on the problems [16]. Let p decision variables make up the vector x. The definition of an optimization problem is:

(1) $$Minimize/Maximize\;f_{i\;}\left(x\right)for\;i=\left(1,2,\;\ldots ,\;n\right)$$

(2) $$s.t.g_{j}\left(x\right)\leq 0\;for\;j=\left(1,2,\ldots ,J\right)$$

where $x=\left\{x_{1},x_{2},\ldots ,\;x_{p}\right\}$ and each $x_{i}$ denotes the *i*-th decision variable (*i*-th alternative). Such optimization problems can be solved using a variety of techniques provided in mathematical programming tools. If the PdM is concerned with the economic aspects of resource management, cost-benefit analysis is frequently used as its optimal criterion [17]. 

As visualization technology develops and becomes more widely used, several novel data-driven PdMs have been adopted to address human and organizational factors. However, this technology has not yet been properly reviewed in the literature. Therefore, the objective of this systematic literature review (SLR) is to synthesize existing evidence regarding the use of PdM in domains such as utilities, power generation, manufacturing, and energy consumption. Our interest is in papers where visualization aids from PdM are used by the personnel to perform some professional tasks. 

## 2. Research Methodology

### 2.1. Research Questions (RQs)

Research questions help us to conduct research and analyze data effectively. The questions listed below are reviewed and answered in detail in Section 3.

RQ1. What are the challenges faced by the current techniques or algorithms (data-driven techniques) for PdM of different utilities?

RQ2. How is the visual analysis of PdM used during the training and evaluation of PdM?

RQ3. What knowledge gaps exist in the PdM field, and what obstacles must be overcome in PdM research before visual analytics can fully complement data-driven techniques?

### 2.2. Search Strategy

The three electronic databases used to find relevant papers for this systematic review were Scopus, Web of Science (WoS), and IEEE Xplore. A brief exploratory study was conducted to determine the suitable search phrases, which resulted in the terms “predictive maintenance” and “machine learning” or “data visualization”. In order to focus our research on works that utilized predictive maintenance and visual analytics in the manufacturing sector, a second set of terms: “smart manufacturing” and “manufacturing ecosystem”, was also identified. To guarantee that no relevant work was omitted, other versions of these terms, such as “smart factories” and “advanced manufacturing”, were also used.

### 2.3. Inclusion Criteria

Studies that utilized PdM with the purpose of optimizing utilities, power production, manufacturing, and energy consumption, as well as being published in journals or conference proceedings, were considered for inclusion. Studies published as posters, dissertations, theses, abstracts, or commentaries were not included. Only papers written in English were considered. The year filter was applied to select the primary studies published from 2017 to the present. To leverage PdM in Industry 4.0, studies that discussed human-centered data visualization methods were included. Papers that described the use of PdM for transport operations, oil, and gas mining were excluded because they fell outside the scope of this systematic review. Moreover, only papers that presented implemented systems were included; publications that merely presented guidelines or protocols, architecture, or theory of a system were excluded. Moreover, this SLR excluded guidelines, review articles, case reports, letters to the editor, and editorials. In terms of visual analytics, studies that merely show exploratory data analysis (EDA) without any feature learning or feature engineering were omitted in this SLR. 

### 2.4. Assessing Study Relevance

The relevant studies were assessed by excluding studies that scored below an established ‘quality threshold’. This is used to differentiate the studies in terms of overall contribution. It can also be utilized to obtain a better grasp of the pros and cons of the body of evidence, which can be taken into consideration throughout the synthesis process [18]. The quality assessment technique used in this SLR is based on the following weighted parameters, presented in [19].

- Novelty: Is the proposed concept truly original, or is it just an improved version of an existing concept?
- Content and Analysis: Was the information presented technically sound and supported by ideas and data that had comparative advantages over state-of-the-art methods?
- Results: When compared to the benchmark data set, was the result presented clearly?

A score out of 10 was assigned to each study. The score was distributed as follows: 2 for novelty, 4 for content and analysis, and the remaining 4 for outcomes, including 2 for dataset and 2 for evaluation criteria (Table 1). 

The number of papers that were eventually included in the review decreased as a result of the adoption of quality rating criteria. The final selection for this SLR excluded studies that were judged to be weak or below average.

### 2.5. Data Extraction

The objective of this stage was to gather information from the selected studies and identify which ones addressed which research question. To do this, a mapping exercise was conducted. From the selected papers, the information extracted from the literature was the author, year, publisher, technique, dataset, PdM type, details and scope, and outcome of the proposed method.

### 2.6. Data Synthesis

To acquire pertinent data for the research questions we sought to address from the articles, synthesizing the data into a table was a necessary step in the data collection and synthesis procedure (Table S1: Results Table of the SLR). In terms of data, we gathered the techniques or algorithms used for PdM, the dataset used, the type of PdM, details and scope of the study, and lastly, the results and solutions they achieved. 

In this SLR, we tabulated the extracted data and then used pie charts to illustrate it. In brief, we began the research process in this SLR by applying search terms to 3 e-databases identifying 964 papers. Out of 964 studies, 672 remained after removing duplicates. Subsequently, 112 studies were identified as appropriate for further analysis after applying the inclusion and exclusion criteria. Only 37 studies met the quality assessment criteria. The review’s final result was summarized to answer all RQs, identify research gaps, and suggest future paths. 

## 3. Results

**Figure 2:**
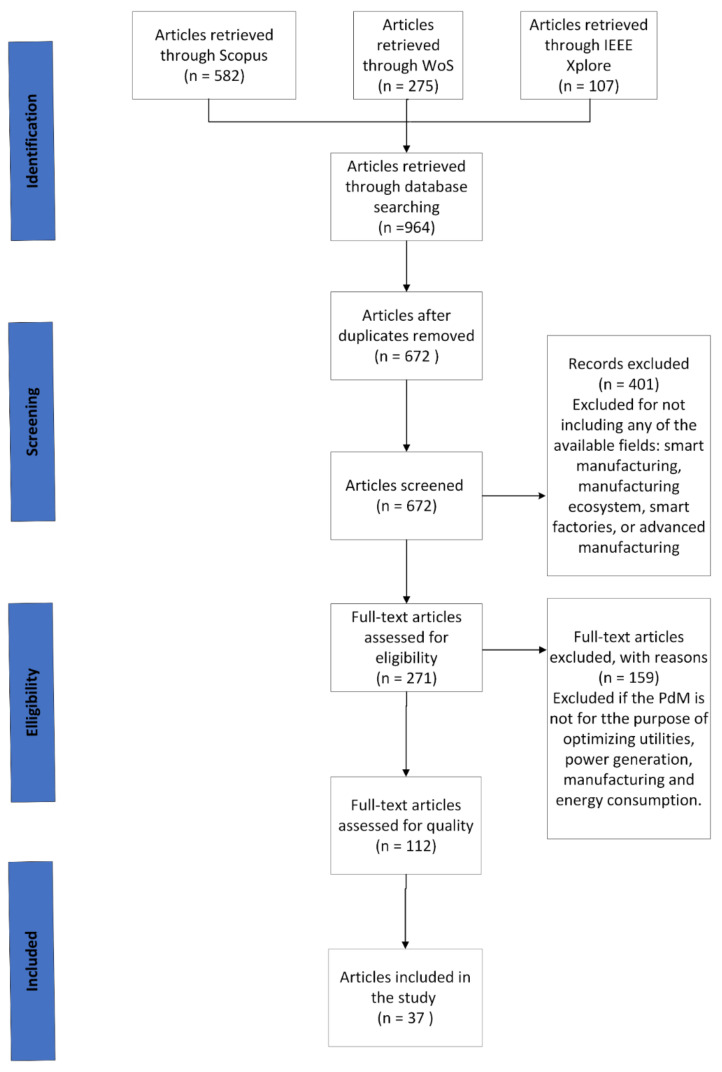
Search flow based on the PRISMA guidelines.

A total of 37 studies were included in this SLR after conducting a search flow based on the Preferred Reporting Items for Systematic reviews and Meta-Analyses (PRISMA) guidelines [20]. Figure 2 shows the review process adopted in this SLR.

### 3.1. Techniques or Algorithms Required for PdM of Different Utilities

**Figure 3:**
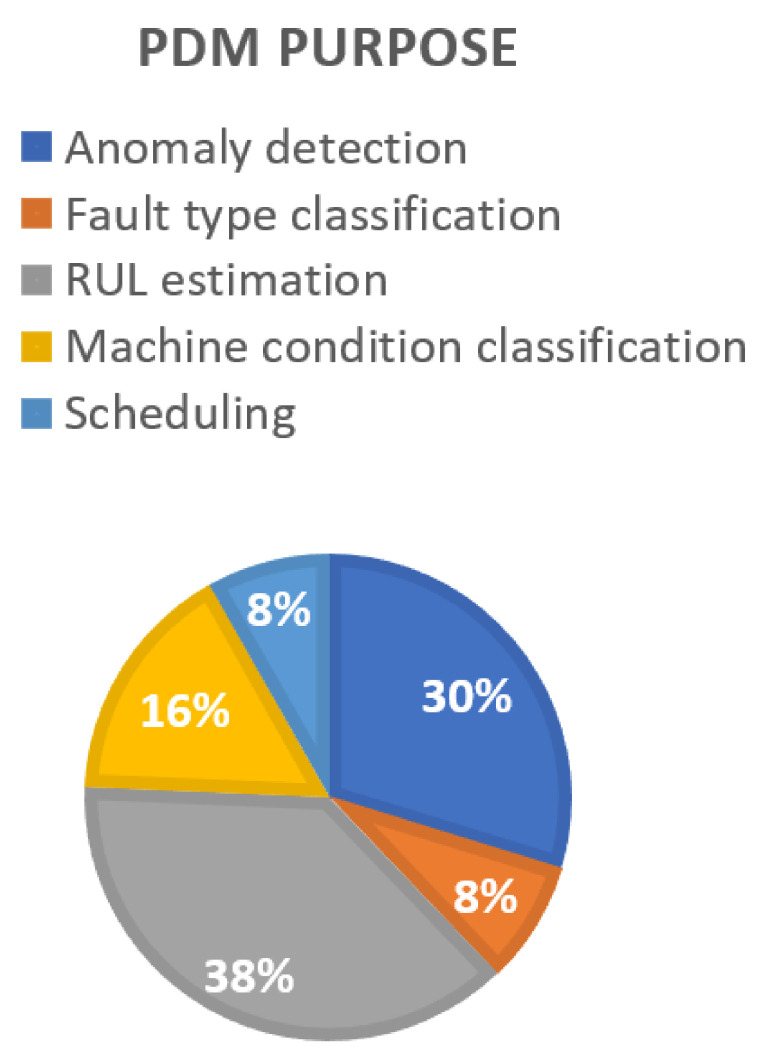
The purposes of PdM in the reviewed works.

Our review showed that estimating remaining useful life (RUL) was the most common purpose in the PdM work. Of the 37 studies, 14 used PdM for estimating RUL, 11 for anomaly detection, 6 for machine condition classification, 3 for fault type classification, and the remaining 3 for scheduling (Figure 3).

Table 2 shows the mapping of PdM techniques and the studies using them. It is obvious that a variety of techniques ranging from feature learning based to machine learning, and to the recent deep learning based, have been used in PdM. From Table 2, conventional machine learning techniques include decision trees (DT), artificial neural networks (ANN), random forests (RF), Naïve Bayes (NB), support vector machines (SVM), principal component analysis (PCA), or ensemble learning. Deep learning includes convolutional neural networks (CNN), recurrent neural networks (RNN), and long short-term memory (LSTM). The detailed techniques are presented in the Table S1 of the systematic review. 

**Figure 4:**
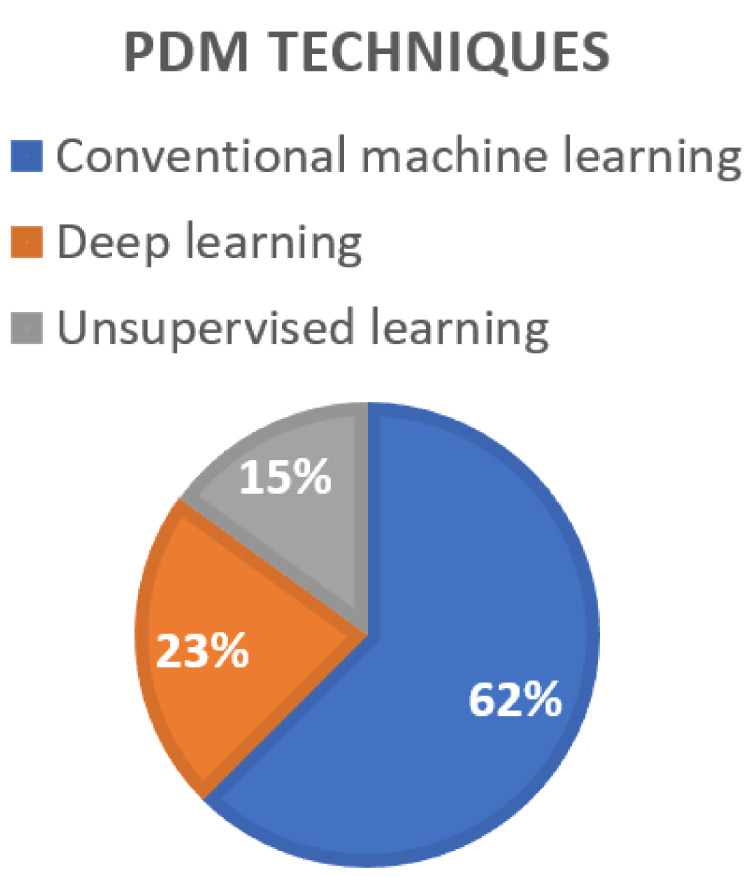
The techniques of PdM in the reviewed works.

Figure 4 shows that conventional machine learning has been a preferred technique, with 62% usage in PdM. This is due to its simplicity of implementation and the availability of off-the-shelf models. Next is the deep learning technique with 23% because of its robustness to noise as long as the models are trained with high data quality. However, it is challenging to acquire labeled data of high quality. Thus, K-means clustering is always used to expedite the labeling process when it comes to anomaly detection. 

From a technical perspective, the CNN structures in different studies have their own characteristics to fit the problem sets. Of all the included studies that used Convolutional Neural Networks (CNN), 3 to estimate the RUL and 1 to detect anomalies in time series data, 9 studies reported satisfactory performance of machine condition classification by using conventional machine learning methods. 

Combining Industry 4.0 with the growth of artificial intelligence (AI) and the emergence of the Internet of Things is paving the way for the full digitization and automation of industrial processes. Resende et al. [52] designed the TIP4.0 (based on the edge of the gateway modular software solution for the PdM platform), using the convolution neural network (CNN) architecture, which highlights the distributed mass edge computing ability in the industrial scene. Another author has proposed an experimental framework to trigger effective PdM for conveyor motors in small-scale manufacturing, using classification models established through time series input data imaging and CNN [45]. 

Data acquisition plays a vital role in PdM in the manufacturing industry. A method based on outlier identification was proposed to reduce, on average, 90.25% of false positives during prediction [27]. To optimize the data management of sensors, a Supervisory Control and Data Acquisition (SCADA) system is integrated with various machine learning algorithms to improve the efficiency and accuracy of predictions. 

Additionally, PdM of production lines is also important to early detect possible defects and thus identify and apply the required maintenance activities to avoid possible breakdowns [56]. The authors developed a new RUL prediction method by utilizing a principal component analysis (PCA) feature selection algorithm, a grid search parameter optimization algorithm, and a multi-layer perceptron (MLP) machine learning algorithm. The NASA dataset was used to evaluate the new model. The results show that RUL can be accurately predicted under a single data attribute. However, in the real production environment, it is difficult to obtain single environmental data, and the prediction effect needs to be improved.

Overall, machine learning plays an important role in PdM, showing good prospects in the manufacturing industry. To perform PdM more effectively, the operating personnel need a visualization tool to better interpret data patterns.

### 3.2. Visual Analytic of PdM

**Figure 5:**
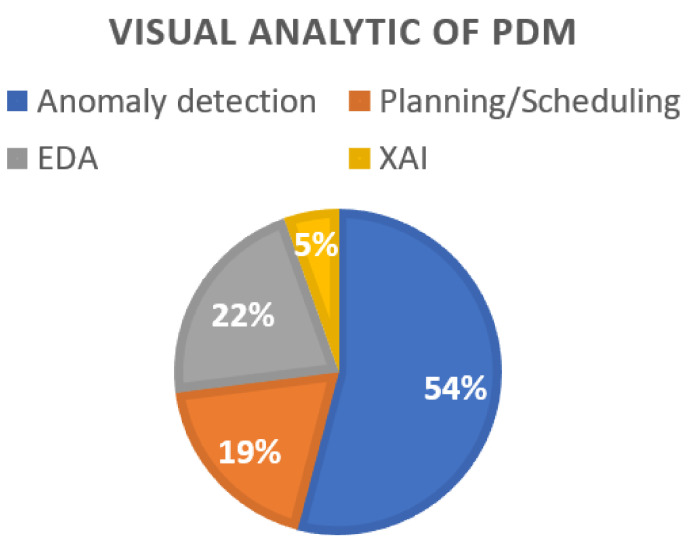
Visual analytic purpose of PdM in the reviewed works.

Figure 5 shows the visual analysis purpose of PDM in the 37 studies, among which anomaly detection accounts for 54%, planning/scheduling accounts for 5%, EDA accounts for 22%, and XAI accounts for 19%. Data visualization is an important tool in data analysis because it allows practitioners to visually recognize complicated data structures and trends. To uncover the most informative data projections it always boils down to data analysts providing the same level of insight from EDA. This SLR has omitted many studies that did not employ visual analytics at the PdM final stage. Thus, Figure 5 shows that the highest percentage belongs to anomaly detection instead of EDA. In fact, it is common to utilize visual analytics at the EDA stage, which is not the scope of this SLR. 

Only a few studies incorporated visual analytics into planning or scheduling so that the domain expert’s expertise was taken into consideration when determining the representative and discriminative features for PdM. However, most of the studies did not integrate the visualization layer for the end practitioners to provide feedback for PdM system improvement. Again, visual analysis was done at the EDA stage only.

The following related works were elaborated in this SLR due to their high relevance and quality.

1. Anomaly detection. Wenjin Yu et al. [14] used fault detection in PdM where a four-layer architecture was presented, consisting of big data intake, management, analytic, and visualization levels, with functionalities ranging from Internet-of-Things (IoT) data acquisition to real-time system condition monitoring. The importance of visual analytics was highlighted here as it extended until the monitoring stage, where the engineer was responsible for monitoring the condition of the compressor even if an anomaly was detected. A 5-minute deterministic mechanism was implemented to set the timestamp from ‘0′ to ‘1′ if more than 15 anomalies out of 300 observations were detected in every 5-minute window. The involvement of an engineer could reduce the possibility of false alarms.
2. Planning/scheduling. From the planning and scheduling perspective, machine learning (ML) with real-time data acquisition can effectively forecast the causes of manufacturing disruptions, which allows the personnel to respond to the changes on the shop floor in a more timely and cost-effective manner. Eman Azab et al. [34] included the PdM timeslot predicted by the optimal machine learning model in the production schedule, which was then visualized in Gantt chart format. Simon Zhai et al. [49] involved maintenance personnel only once to select relevant operational parameters for PdM. Then, K-means clustering was adopted to group the operational conditions. The clusters served as the response variables for training a Health Indicator model. The output of this model would be integrated into scheduling algorithms which are not elaborated on in this study.
3. EDA. PdM promotes the use of machine learning technologies to track asset health and schedule repairs accordingly. Informative features are required to train an effective machine learning model. Hence, thorough EDA is normally performed before the modeling process. This is because effective predictive modeling necessitates a number of stages, but the interpretability of the results may become harder to grasp and less generalizable in the latter stages. Different models have respective metrics to present the feature importance. For example, the metric used to show the feature importance of logistic regression is the coefficients found for each input variable [57], while the decision tree looks for the decrease in node impurity weighted by the probability of reaching that node [58]. In contrast, EDA could be the gold standard methodology to analyze a dataset due to its generalizability, such as correlation scores or pair-plot [59].

Therefore, most of the PdM studies involved maintenance personnel at the EDA stage only to perform manual feature engineering. Nevertheless, it is challenging to deliver high-quality feature engineering that can be used for a variety of PdM applications. As a result, a more automated feature engineering method is required. Semi-supervised learning [21,47,53,54] was the most common feature learning approach for PdM. Pooja Vinayak Kamat et al. [47] used the K-means technique with Silhouette Coefficient for anomaly trend analysis after EDA was performed to rank the feature importance. Then, a deep learning model that used the autoencoder-LSTM (AE-LSTM) technique was applied to classify both normal and abnormal clusters generated previously. The common issue faced by these studies is the inability to validate the model’s decision-making by comparing it to common knowledge or maintenance personnel’s experience. 

Compared with other PdM architectures that adopted a feature learning approach, a more comprehensive study was conducted by Antonio L. Alfeo et al. [13] to improve its interpretability. They proposed feature learning, in particular for each input signal from different sensors and domain transformation, giving some insight into their role in the classification model. They implemented this by utilizing K-means clustering to achieve the best quality combination of features for a given classification task. Consequently, this study provides better interpretability of the learned model, and the maintenance personnel could relate the PdM to the measure analyzed.

4. XAI. Steenwinckel et al. [22] integrated data-driven techniques with knowledge-driven techniques in PdM, allowing it to complement the weaknesses of one with the strengths of another and, therefore, improve the interpretability of an ML model with optimized performance. Although the PdM’s purpose of this study was not in the manufacturing industry, it was included in the SLR due to its significant results in showing the importance of incorporating context-aware alerts to the operating personnel. The authors proposed to fuse domain knowledge in every phase of methodology in their study. In the first phase, i.e., the EDA stage, the outputs of the data- and knowledge-driven techniques were merged into a semantic database so that the detected anomalies were accompanied by reasons for why they happened. In the second phase, a dashboard visualization was created to let users provide feedback on the anomalies by indicating their accuracy or relabeling them based on an expert’s knowledge. The third phase is the optimization stage, where anomalies were detected by using all the background knowledge, context information, anomalies, causes, and feedback maintained in the semantic database. Even at this stage, the visualization enables a continuous feedback loop between the detected events and their cause based on the semantic database.

## 4. Discussion

The goal of this SLR was to review the literature on the use of visualization aids for PdM in the manufacturing industry. After 37 articles that satisfied the defined criteria were compiled, a table (Table S1) that contains the relevant information for answering the research questions was created. Next, the mapping of the studies with the RQs is presented, followed by a discussion of our findings. 

RQ1 intended to discover the challenges faced by the current techniques or algorithms used for PdM of different utilities. A few were discussed in Section 3.2. PdM approaches can be divided into two categories which are data-driven and knowledge-driven techniques. Data-driven techniques encompass pre-processing steps to transform the data collected from sensors into a set of useful features. Subsequently, machine learning models can be trained by using the features. In contrast, knowledge-driven techniques require domain and background knowledge to accurately identify the true causes of anomalies, which commonly involve human experts. RQ1 addresses data-driven techniques, while visual analytics is used to address RQ2, which incorporates knowledge-driven techniques. The challenges encountered by the PdM approaches are as follows.

1. The PdM approach lacks the ability to generalize. This is because the degradation processes vary widely across industries, facilities, and machines. Furthermore, the PdM approaches are designed and customized to specific problems based on the data collected from the sensors to assess the asset’s health condition. Hence, different machines in the same manufacturing factory may require an individual PdM approach to achieve effective results.
2. To achieve generalizability, PdM approaches collect temperature, vibration, power consumption, and noise from different machines. However, PdM approaches are dependent on the type of degradation. It would require different measures to detect partial breakage or deterioration of a component or the asset’s operating condition in which some measures may be less informative than others.
3. To ensure that each measure is properly treated, the PdM approaches engage both data scientists and maintenance personnel in the framework to perform manual feature engineering. Another significant aspect of manual feature engineering is the high management cost because this process may be repeated during the architecture training and evaluation to achieve better performance. As a result, most of the studies involved the maintenance personnel until the EDA stage only. Some of the studies [44,45,46,47,48,49,50,51,52] adopted a more automatic feature engineering process, such as CNN.
4. The deep learning approach, such as CNN, was often used to achieve higher performance for PdM. Nevertheless, this black-box approach only allows the prominent display of input and output parameters while hiding the intrinsic relationships between them. By using this approach, the features are obtained as a non-linear combination of the inputs, making it difficult to grasp the contribution of the inputs to the classification output and, hence, the model’s logic. In real-world applications, such as industrial manufacturing processes, it is preferable to prevent such a lack of transparency. This is because PdM applications may involve important decision-making that requires the practitioner’s feedback. It can only be more reliable to have some justifications behind the individual prediction made by an AI algorithm, particularly in an automated setting.

RQ 2 intended to discover how visual analytics have been used in PdM. The literature survey presented in Section 3.2 summarizes the different types of visual analytics used in the studies included in this review. The researchers used visual analytics at different stages of the PdM framework. Figure 5 shows the purpose-wise distribution of studies using visual analytics. It is seen that EDA was explored more as compared to others due to it being an essential step of the PdM framework. Only EDA that leads to feature learning or engineering is included in this SLR. XAI is the next choice to optimize PdM. The following are the ways visual analytics are employed in PdM.

1. **Anomaly detection**. Undoubtedly, machine learning models are booming in PdM applications. They have shown promising results in anomaly detection, where they could predict machine failures or RUL via measurement from sensors, unknown or abnormal patterns, and events. The drawback is that small, inconsistent anomalies may occur that do not reflect the real condition of the machine [14]. Visual analytics allows domain experts to point out redundant anomaly detection, thereby improving the performance of PdM.
2. **Planning/scheduling**. After PdM is employed, the health indicators or degradation indicators could help maintenance personnel better plan the maintenance operation. By optimizing the maintenance schedule, unnecessary maintenance and high prevention costs can be avoided [60].
3. **EDA**. To gain a better insight into the data acquired, an unsupervised clustering technique (i.e., K-means) was often implemented at the EDA stage to discover if the resulting clustering labels matched the order of the actual health stage labels. Scatter plots are then presented as a group of points where those that follow the same general pattern are visualized as the same cluster and vice-versa for those that have different patterns.
4. **XAI**. Visual analytics was used as an aid to realizing XAI. The outcome provided by the machine learning models could be justified by interpretable XAI techniques such as Shapley values [61]. The Shapley values are able to demonstrate which features are significant for the machine learning model or even relate the decision it made to the specific parts of the input. What is interesting in this SLR is the low adoption of such techniques in PdM despite its high interpretability.

Visual analytics was often employed to integrate data-driven techniques with knowledge-driven techniques. This is because it can overcome the respective limitations. The weak interpretability of data-driven techniques may lead to many false positives. On the other hand, knowledge-driven techniques are unable to learn new anomalies automatically and require significant human effort to maintain, even though they have low false positive rates. Despite this, there are currently no standard quantifiable measures to evaluate the quality of certain visualizations. 

RQ3 intended to discover the various usages of visual analytics that could realize effective PdM instead of stopping at the prediction stage without incorporating it into a proper framework. Given that, we decided to make one subsection (Section 4.1) to address RQ3 before the conclusion because RQ3 includes the gaps that are identified after addressing RQ1 and RQ2. 

### 4.1. What Knowledge Gaps Exist in the PdM Field, and What Obstacles Must Be Overcome in PdM Research before Visual Analytics Can Fully Complement Data-Driven Techniques?

One of the common challenges is the lack of labeled failure data in the manufacturing industry. To cope with this challenge, an unsupervised learning approach, especially K-means clustering, is always adopted to accurately find anomalies without the need for labels [22] because collecting all types of failure data in a large-scale monitored factory is impossible. Still, many erroneously introduced alerts or missed interventions may occur if expert knowledge is not taken into consideration. Therefore, visual analytics plays an important role in integrating knowledge-driven techniques into the unsupervised labeling process. 

Therefore, a hybrid or semi-supervised learning approach has been proposed. Humans are much better at detecting anomalies by studying graphs or charts. Therefore, projection techniques are considered a promising approach [33] to visualize patterns by using the spatial coordinates of the original datasets. This common approach is called Exploratory Projection Pursuit (EPP) [62]. Combining EPP with the clustering method could yield informative and intuitive depictions. Subsequently, the maintenance personnel only need to analyze the obtained visualization.

We have identified the gaps that exist in previous studies as follows.

1. Due to the different nature of machines, a custom Data Acquisition (DAQ) device can be embedded into the machines to realize a framework that can process the data via the Digital Twin of the equipment for the calculation of the RUL of critical components.
2. To leverage the benefits of data-driven techniques, knowledge-driven techniques of maintenance personnel have to be included in the framework of PdM in the manufacturing industry. Visual analytics should not be only at the EDA stage but also at the PdM final stage, where the maintenance personnel could provide feedback to improve the operational efficiency of machines.
3. The Augmented Reality (AR) module can be implemented for PdM. An AR module can be installed to make the process of monitoring industrial equipment easier. This module can be implemented as a multi-platform application from which clients can view critical information about their equipment and interact with it quickly and intuitively by using cutting-edge digital technology, whether remotely or on-site.
4. Using the sensor data to train a health model for each component can result in a more interpretable health indicator for PdM and help locate possible failure reasons, such as components with low health indicator values. This is because aggregating component-specific sensor data may prevent the sensor data of two independent, unrelated components from contradicting the overall health indicator trend and canceling each other out. Consequently, the individual health indicator is projected to perform better in terms of evaluation metrics.

## 5. Conclusions

This systematic review aimed to synthesize existing evidence regarding the use of PdM in the manufacturing industry. After exploring the literature, we conclude that there is a significant dearth of conclusive research on involving maintenance personnel’s feedback in the latter stage of PdM architecture, i.e., model building or even model deployment to improve the PdM performance. The role of maintenance personnel remains at the EDA stage in most of the studies. 

With regard to the research questions that we set out to address in this SLR, the integration of data-driven and knowledge-driven techniques would definitely improve the reliability of the PdM architecture and reduce false positive alarms. Moreover, with visual analytics of PdM that are shown, preferably on the dashboard, the maintenance personnel can better react and pinpoint possible solutions for preventive measures. Although PdM has been an active research area with significant algorithm development over the past decades, the most common challenges are the lack of labeled failure data in the manufacturing industry.

Generally, the studies examined in this SLR are considered to be insufficient to establish the impact of visual analytics with maintenance personnel’s feedback on PdM. Given what we currently know, the feedback can be used to supplement any kind of PdM to improve the model continuously. Nevertheless, there are still issues to be resolved before we see PdM utilized seamlessly in the manufacturing industry in terms of maintenance scheduling.

## Figures and Tables

**Table 1:** Quality Assessment.

Quality Level	Number of Studies	Percentage (%)
Good (7 < score <= 10)	7	6.25
Average (5 <= score <= 7)	30	26.79
Poor (score < 5)	75	66.96

**Table 2:** Mapping of techniques with respective studies using them.

Technique Used	Study Number
Conventional machine learning	[13,14,21,22,23,24,25,26,27,28,29,30,31,32,33,34,35,36,37,38,39,40,41,42,43]
Deep learning	[44,45,46,47,48,49,50,51,52]
K-means clustering	[21,33,47,53,54,55]

## Supplementary Materials

The following supporting information can be downloaded at: https://www.mdpi.com/article/10.3390/s22176321/s1, Table S1: Results table of the SLR.
